# Supplementary material for: When the Whole Is Less Than the Sum of Its Parts: Maximum Object Category Information and Behavioral Prediction in Multiscale Activation Patterns
Source: Front Neurosci. 2022 Mar 2;16:825746. doi: 10.3389/fnins.2022.825746 (PMC8924472; doi:10.3389/fnins.2022.825746)

Supplementary Figure 1

Timing and amplitude parameters extracted from the time-resolved accuracies (Figure 2) of each FS algorithm and each dataset and their Bayesian evidence analyses. (A-D) Left: the maximum and average decoding accuracies, the time of maximum and the first above-chance decoding. Bottom section on A and B show the Bayes factor evidence for the difference of the decoding accuracy compared to chance-level decoding; Right: matrices compare the right parameters obtained from different features. Different levels of evidence for existing difference (moderate 3<BF<10, Orange; strong BF>10, Yellow), no difference (moderate 1/10<BF<1/3, light blue; strong BF<1/10, dark blue) or insufficient evidence (1<BF<3 green; 1/3<BF<1 Cyan) for either hypotheses. Black and red boxes show moderate or strong evidence for higher decoding values for specific features compared other sets of features as explained in the text. The horizontal dashed lines on the left panels of (A) and (B) refer to chance-level decoding. Filled circles in the Bayes Factors show moderate/strong evidence for either hypothesis and empty circles indicate insufficient evidence.

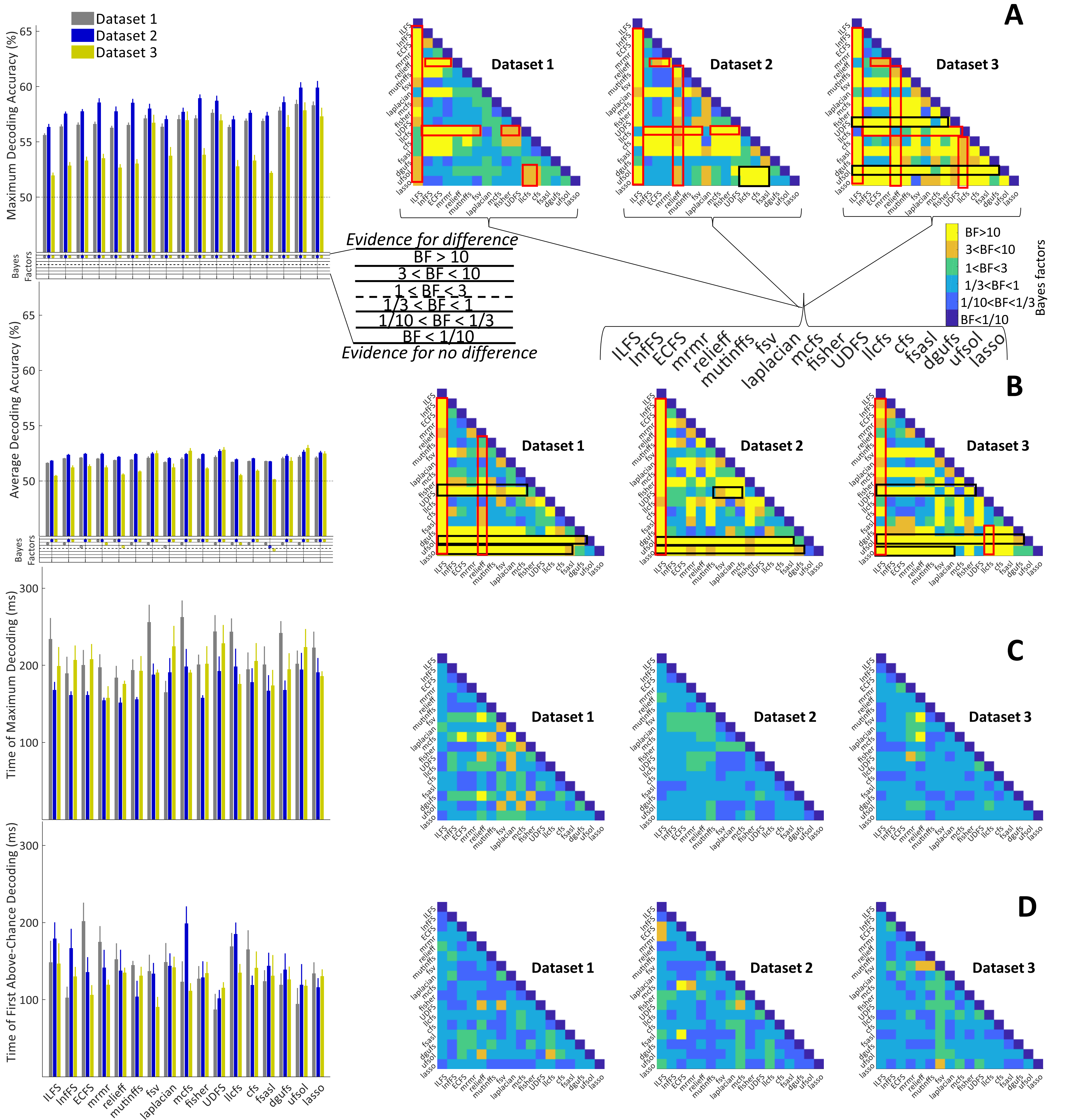

Supplement: Supplementary file 1 [file Presentation_1.pdf]
