# Supplementary material for: When the Whole Is Less Than the Sum of Its Parts: Maximum Object Category Information and Behavioral Prediction in Multiscale Activation Patterns
Source: Front Neurosci. 2022 Mar 2;16:825746. doi: 10.3389/fnins.2022.825746 (PMC8924472; doi:10.3389/fnins.2022.825746)

Supplementary Figure 2

The merit of individual features when combined to maximize the decoding accuracies using 17 different feature selection methods. Warm colors indicate higher merit and cold colors indicate lower merit for the feature at the indicated time point across the trial. Each of the three columns shows the results for one dataset. Merit is the richness of information in the feature about object categories.

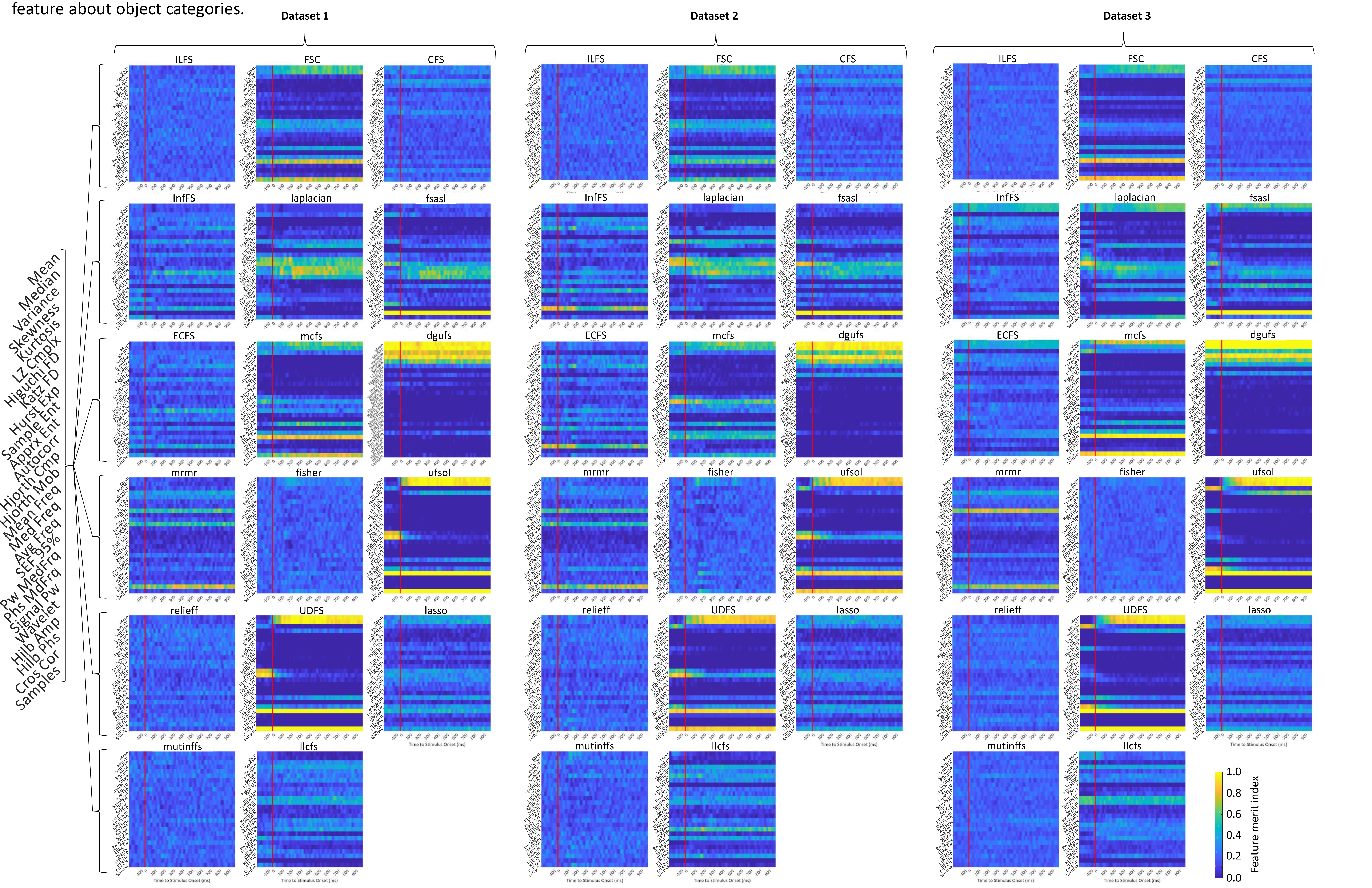

Supplement: Supplementary file 2 [file Presentation_2.pdf]
